# Do patients’ preferences prevail in hospital selection?: a comparison between discrete choice experiments and revealed hospital choice

**DOI:** 10.1186/s12913-022-08403-6

**Published:** 2022-09-08

**Authors:** Benjamin H. Salampessy, David Ikkersheim, France R. M. Portrait, Xander Koolman

**Affiliations:** 1 Department of Health Sciences, Faculty of Science, Vrije Universiteit Amsterdam, De Boelelaan 1085, 1081 HV Amsterdam, The Netherlands; 2 KPMG, Laan van Langerhuize 1, 1186 DS Amstelveen, The Netherlands

**Keywords:** Discrete choice experiment, Patient preferences, Choice behavior, Hospital, Quality of care, Revealed and stated preferences

## Abstract

**Background:**

In patient choice, patients are expected to select the provider that best fits their preferences. In this study, we assess to what extent the hospital choice of patients in practice corresponds with their preferred choice.

**Methods:**

Dutch patients with breast cancer (*n* = 631) and cataract (*n* = 1109) were recruited. We employed a discrete choice experiment (DCE) per condition to measure stated preferences and predict the distribution of patients across four hospitals. Each DCE included five attributes: patient experiences, a clinical outcome indicator, waiting time, travel distance and whether the hospital had been recommended (e.g., by the General Practitioner (GP)). Revealed choices were derived from claims data.

**Results:**

Hospital quality was valued as most important in the DCE; the largest marginal rates of substitution (willingness to wait) were observed for the clinical outcome indicator (breast cancer: 38.6 days (95% confidence interval (95%CI): 32.9–44.2); cataract: 210.5 days (95%CI: 140.8–280.2)). In practice, it was of lesser importance. In revealed choices, travel distance became the most important attribute; it accounted for 85.5% (breast cancer) and 95.5% (cataract) of the log-likelihood. The predicted distribution of patients differed from that observed in practice in terms of absolute value and, for breast cancer, also in relative order. Similar results were observed in population weighted analyses.

**Discussion:**

Study findings show that patients highly valued quality information in the choice for a hospital. However, in practice these preferences did not prevail. Our findings suggest that GPs played a major role and that patients mostly ended up selecting the nearest hospital.

**Supplementary Information:**

The online version contains supplementary material available at 10.1186/s12913-022-08403-6.

## Introduction

In the last decades, many countries have implemented elements of provider competition in their health systems to stimulate effective price and quality competition [1, 2]. This implies that patients – and those who act on their behalf such as health providers and procurers - are expected to make tradeoffs between the price and quality of care and select the provider that best fits their preferences [2, 3]. In theory, patient choice should lead, among other things, to a higher quality of care and lower cost per unit of care delivered. For example, higher-quality providers will attract more patients compared to those with poorer quality who, in turn, are forced to either improve their quality of care or leave the health care market [4].

However, the literature suggests that, in real-life settings, patients are less inclined to take on the role of rational autonomous consumer [5, 6]. Most research has focused on the choice of health care institutions such as hospitals and has either used stated choices (i.e., hypothetical or potential situations in questionnaires) or revealed choices (i.e., real situations), while research that has compared both stated and revealed choices from the same sample remains scarce [6]. Reviews conclude that, in general, patients rely on the advice of their referring physicians such as the General Practitioner (GP) for their choice of hospital [5, 6]. Other important determinants of choice often described in the literature relate to the patient-hospital travel distance or the presence of any previous experiences patients may have had with a particular hospital. However, these factors are considered to be of less importance than the GP’s advice [5–7]. In addition, although quality information has increasingly been made available publicly, patients are often neither aware that such information is offered nor that levels of quality may differ across providers [6]. More importantly, on the occasions that patients do use quality information to choose providers, they do so selectively [7, 8].

Although the choice for providers seems still to be largely determined by the referring GP [5, 6], this does not have to be a problem. If the GP acts as a perfect agent – i.e., considers the preferences of his or her patient when making the referral - then patients would still be referred to the provider that the patient would have proactively chosen otherwise. However, research has shown that physicians are often not aware of what patients really want when making health care decisions, and that quality information plays a limited role when conducting their referrals to hospitals [9–11]. Consequently, the assumption made in many current health policy reforms – i.e., that patients and those who choose on their behalf, choose hospitals in accordance with the preferences of patients – may not in practice be so.

This study assesses to what extent a patient’s choice of hospital in real-life settings corresponds with their preferred choice. More specifically, we have first estimated the stated preferences of Dutch patients (breast cancer and cataract) regarding hospital characteristics, then used the estimated preferences to predict the distribution of patients across hospitals. Subsequently, we have compared this distribution to that observed in real-life settings. As we have collected stated and revealed choice data from the same patient sample, the main contribution of our study is to add new evidence to the limited literature that has compared both stated and revealed choices from the same sample [6].

The Dutch context is highly suitable for this type of studies for several reasons. First, similar to the US, provider competition has been implemented in the Dutch health system since 2006. The reform aims at stimulating effective competition between providers on price and quality and at encouraging patients to take an active role in health care decisions. Second, universal access enables Dutch patients to use care the cost of which is (to a large extent) covered by the basic health insurance package which includes, for example, hospital care and maternity care. GPs act as gatekeepers for most care covered by this package [2, 8]. Last, to encourage patient choice, comparative information is presented for a large variety of conditions via online platforms (for an example, see [12]).

## Methods

### Study population

We focused on insured individuals who had received surgery for breast cancer or cataract in 2010 in one specific region of the Netherlands. We chose these conditions for two main reasons. First, as publicly reported quality information was only available for a small number of conditions in 2009, we selected breast cancer or cataract since the quality information had been collected for a relatively large number of patients compared to, for example, diseases of the adenoids and tonsils. Second, breast cancer and cataract patients had to exercise choice in very different settings, given the differences in medical urgency for treatment. In 2010 and across all Dutch hospitals, the waiting time between referral and first hospital appointment was, on average, 1.5 weeks for breast cancer and 4.9 weeks for cataract [13].

Patients were identified using claims data of 2010 and were eligible if they had received surgery in one of the four general hospitals located within a 15-km radius of the metropolitan area of Eindhoven in the Netherlands. This area had 750,000 inhabitants in 2010 [14] and was considered to be highly suitable due to the absence of academic hospitals as this would avoid potentially undesirable concentrations of high-risk patients (e.g., patients with comorbidities) which generally occur in these hospitals.

### Discrete choice experiments

To measure stated preferences, we conducted discrete choice experiments (DCEs). In this methodology, respondents are presented with a series of hypothetical scenarios (choice sets). Each choice set contains two or more alternatives that are described by a systematic combination of attributes and levels. Within each choice set, respondents indicate their most preferred alternative which, according to the Random Utility Maximization framework [15], is assumed to maximize their utility thereby reflecting their latent preferences. In our study, patients had first received their surgery and were then invited to complete the DCE questionnaire (i.e., post-surgery). We conducted a DCE for each condition separately.

#### Attributes and levels

To select attributes, several relevant factors with regard to a patient’s choice of hospital were identified in the literature. We included two quality indicators as attributes: (1) patient experiences and (2) clinical quality. For the former, we used the hospitals’ scores on the Dutch Consumer Quality Index (CQI) questionnaire (i.e., a translated and validated version of the Consumer Assessment of Healthcare Providers and Systems implemented and used in the US [16]). For the latter, a shortlist was first created from the full set of indicators (i.e., as published publicly for reporting year 2010 [17]), and then discussed with GPs who identified an outcome indicator as the most important one. This selection procedure had been performed as part a previous study and was therefore conducted by Ikkersheim and Koolman [10]. In addition to these quality indicators, we included three other factors as attributes: (3) waiting time, (4) travel distance to the given hospital and (5) a measure identifying the person who had recommended the given hospital to the patient. Accordingly, each DCE included five attributes each with three levels (Table 1). Levels for waiting time and clinical outcome indicator were based on the actual distribution in scores to reflect meaningful and realistic levels [17, 18]. The expected sign for each attribute was based on manuals of quality indicators and on common sense.

**Table 1:** Hospital characteristics (attributes) and levels, DCE

Attributes	Stratification	Description	Levels	Expected sign ^**a**^
1) Patient experiences ^b^		Level of satisfaction regarding the attention, explanation and time.	Below average, average and above average	+
2) Clinical outcome indicator	*Breast cancer:* tumor-positive resection margin	Share of resections for which the tumor resection margin was shown to be tumor-positive in the first surgery in breast saving therapy.	5, 10 and 20%	–
	*Cataract:* per-operatively performed vitrectomy	Share of surgeries for which a vitrectomy was performed per-operatively due to a surgery-related complication.	0.4, 0.8 and 1.7%	–
3) Waiting time		Waiting time between the referral and the first hospital appointment.	5, 15 and 40 working days	–
4) Travel distance		Travel distance from the residential home to the hospital.	3, 8 and 15 km	–
5) Recommendation		The person or persons who had recommended the given hospital.	Nobody in particular, friends and family, and GP	+/−

*DCE* Discrete choice experiment, *GP* General Practitioner, *KM* Kilometer

^a^based on the indicator’s manual and on common sense

^b^based on the Dutch Consumer Quality Index questionnaire

#### Experimental design and questionnaire

We followed the principles of Street and Burgess to generate an experimental design [19]. We used the 81-array orthogonal main effects plan (five attributes, three levels each). We applied the fold-over technique by shifting attributes’ levels (modulator: 22222) to create the second alternative. Each choice set consisted of two unlabeled hospitals and asked respondents to indicate which one they preferred; an example choice set is included in Supplementary Material 1. The final design was blocked: seven blocks that contained ten choice sets each and one block that contained eleven choice sets.

In each questionnaire, we fully explained the DCE task and provided an example of a choice set. In addition, we included questions regarding personal characteristics (i.e., age, gender and self-reported general and mental health (only for breast cancer)), education level, whether respondents had received any advice with regard to their choice of hospital and whether respondents had been provided by their GP with quality information to make their choices. We piloted the DCE: minor adjustments in wording were made afterwards, but no changes to attributes and levels were necessary. In 2011, questionnaires were distributed by mail to 1391 breast cancer and 1816 cataract patients. A reminder was sent after four and 8 weeks to those who had not yet completed the questionnaire. Participation was voluntary. All identifying characteristics such as names were replaced with a simple serial number. Hence, it was not possible for the researchers to identify individuals.

### Revealed choices

As we used claims data to identify eligible patients, we could also determine in which of the four hospitals patients had received their surgery (revealed hospital choice). To describe the four general hospitals, we used four of the five attributes as information for recommendation was not recorded in claims data. For waiting time, patient experiences and clinical outcome indicator, we derived the hospital’s actual score on these measures from national databases and included these values as level [17, 18]. For travel distance, we first computed the patient-hospital distance using postal codes and then transformed this distance into an appropriate level also used in the DCE (3, 8 or 15 km).

### Econometric analysis

#### Stated preferences

To analyze our DCE data, we performed mixed logit (MXL) models as these models accommodated for the within-respondent correlation in the data (multiple choice sets per respondent) and allowed for individuals’ preference variation [15, 20]. To estimate utilities, we relied on the Random Utility Maximization framework. Assuming a linear additive utility function, we modelled ‘V_ij_’ using the following equation:

1 $${\mathrm{V}}_{\mathrm{ij}}=\left({\upbeta}_0\right)\ast \mathrm{ASC}.{\mathrm{hospitalA}}_{\mathrm{j}}+\left({\upbeta}_1+{\eta}_{1\mathrm{i}}\right)\ast {\mathrm{Waitingtime}}_{\mathrm{j}}+\left({\upbeta}_2+{\eta}_{2\mathrm{i}}\right)\ast \mathrm{Traveldistance}.8{\mathrm{km}}_{\mathrm{j}}+\left({\upbeta}_3+{\eta}_{3\mathrm{i}}\right)\ast \mathrm{Traveldistance}.15{\mathrm{km}}_{\mathrm{j}}+\left({\upbeta}_4+{\eta}_{4\mathrm{i}}\right)\ast \mathrm{Recommendation}.{\mathrm{friendsandfamily}}_{\mathrm{j}}+\left({\upbeta}_5+{\eta}_{5\mathrm{i}}\right)\ast \mathrm{Recommendation}.{\mathrm{GP}}_{\mathrm{j}}+\left({\upbeta}_6+{\eta}_{6\mathrm{i}}\right)\ast \mathrm{Patientexperiences}.{\mathrm{average}}_{\mathrm{j}}+\left({\upbeta}_7+{\eta}_{7\mathrm{i}}\right)\ast \mathrm{Patientexperiences}.{\mathrm{aboveaverage}}_{\mathrm{j}}+\left({\upbeta}_8+{\eta}_{8\mathrm{i}}\right)\ast {\mathrm{Clinicaloutcomeindicator}}_{\mathrm{j}}$$

In eq. 1, ‘V_ij_’ reflected the systematic part of the latent utility ‘U’ and represented the utility derived by the respondent ‘i’ from the given alternative ‘j’ in a choice set as described by the combination of levels on each attribute. ‘V_ij_’ was captured by an alternative specific constant (ASC), the mean attribute utility weights ‘β_0_-β_8_’ and the individual-specific variation in utility weights ‘ɳ_1_-ɳ_8_’. The ASC was included to account for any left-to-right bias. Waiting time and clinical outcome indicator were coded as continuous variables with a linear specification. Standard dummy coding was used for the remaining attributes. To improve the validity of our results, models were bootstrapped using 250 bootstraps with replacement [21].

To determine the relative importance of attributes, we computed the marginal rate of substitution (MRS) and used waiting time as the denominator. As suggested by Train and Weeks, we computed the MRS measures in ‘willingness to pay’-space in order to obtain more stable MRS estimates [22, 23]. To do so, we re-parameterized the MXL model by rewriting the utility function such that an estimated coefficient reflected the MRS for that attribute.

#### Distribution of patients

We first used the hospital’s levels (as described above, excluding recommendation) and the estimated individual-specific marginal utility estimates to compute choice probabilities for each hospital per respondent. To obtain the predicted distribution across the four general hospitals, we computed the average choice probability per hospital by taking the average of the choice probabilities for that hospital across all individuals. We then compared the expected distribution of patients to that observed in real-life settings in terms of absolute value and relative order. Additionally, as we bootstrapped our MXL models, we performed the aforementioned predictions in each bootstrap iteration (*n* = 250 per condition) to quantify the uncertainty of our point-estimate and compute 95% confidence intervals (95% CI).

#### Additional analyses

As described above, we could only use four of the five attributes to describe the four general hospitals in our predictions. To assess the effect of the omitted attribute, we repeated our main analyses based on four attributes (hereafter referred to as 4-attributes analyses).

In addition, as our patients were recruited from one specific area (i.e., the area of Eindhoven), we performed inverse probability weighted (IPW) models to make our findings more representative of the total patient population [24]. We used iterative proportional fitting to compute the weights to ensure that the weighted marginal totals of the sample closely resembled those of the total patient population (gender and age) and large representative samples (educational level) [25–28].

Moreover, to understand our findings better, we investigated the explanatory power of each attribute based on revealed choice data. More specifically, we calculated the contribution of an attribute to the overall log-likelihood of the full model as described by Lancsar et al. [29]; this procedure is similar to calculating partial R-squares (explained variance, R2)). In short, we first performed a conditional multinomial logit (MNL) model and then re-estimated the model in which we omitted one attribute at a time. Subsequently, we determined the difference in log-likelihood between the full model and that of the reduced model.

All models were estimated using the R-package “Apollo” [30]. Iterative proportional fitting was performed using the R-package “anesrake”. Results were considered statistically significant if the *P* value was < 0.05.

## Results

### Respondents

The questionnaires were completed by 1740 respondents in total (response rate: 46.5% (breast cancer) and 66.6% (cataract)). As shown in Table 2, most respondents were female, aged between 45 and 64 (breast cancer) or between 65 and 79 (cataract), had attained a low educational level and perceived their general health and mental health (breast cancer only) as good to very good. In addition, most respondents had not been provided by their GPs with hospital quality information. While the majority of the cataract group indicated that no one in particular had recommended the hospital in which they received their surgery, proportionally more persons in the breast cancer group relied on the advice of their GP. Furthermore, on average and relative to representative patient populations, respondents (both patient groups) were similar in terms of age and gender but had attained a lower educational level.

**Table 2:** Respondents’ characteristics

	Breast cancer		Cataract	
	Study sample (*n* = 631)	Dutch patients ^a^	Study sample (*n* = 1109)	Dutch patients ^a^
	%	%	%	%
*Demographics*				
Gender				
Male	0.0	1.3	42.9	40.5
Female	100.0	98.7	54.2	59.5
Missing	0.0	0.0	2.9	0.0
Age				
18–44 years	26.0	20.5		
45–64 years	50.7	50.1		
65 years and older	23.3	29.4		
Missing	0.0	0.0		
18–64 years			25.7	18.6
65–79 years			60.2	54.0
80 years and older			14.1	27.4
Missing			0.0	0.0
*Socio-economic status*				
Education level				
Low	44.8	25.1	68.4	40.9
Moderate	33.1	42.6	13.1	36.6
High	16.6	32.3	9.9	22.5
Not-disclosed or missing	5.5	0.0	8.6	0.0
*Self-reported health status*				
General health				
Poor	7.3		1.8	
Moderate	18.7		22.6	
(Very) good	73.4		68.8	
Missing	0.6		6.8	
Mental health ^*b*^				
Poor	15.1		0.0	
Moderate	22.2		0.0	
(Very) good	61.9		0.0	
Missing	0.8		100.0	
*Being well-informed on quality information*				
Patient experiences				
No	76.7		57.7	
Yes	16.5		32.6	
Do not know	5.5		4.5	
Missing	1.3		5.2	
Clinical quality indicators				
No	69.9		47.3	
Yes	23.1		41.9	
Do not know	5.7		5.3	
Missing	1.3		5.5	
*Advice received in hospital choice (self-reported)*				
Who had recommended the chosen hospital				
No one in particular	46.0		55.3	
Friends	4.4		10.6	
GP	45.2		24.3	
Missing	4.4		9.8	

*GP* General Practitioner

^a^source: [25–28]

^b^mental health was only measured for breast cancer

### Stated preferences

Both MXL models (breast cancer and cataract) demonstrated theoretical validity (Table 3). All attributes had their expected sign and levels that were significant. For dummy coded attributes with ordered levels (i.e., patient experiences and travel distance), the ordering of the point estimates was consistent with the ordering in levels; for example, the marginal utility for the “above average” level of patient experience (breast cancer) was larger than that of the “average” level.

**Table 3:** Results of mixed logit models (main analyses)

		Breast cancer			cataract		
		beta	SE ^a^	*P* value	beta	SE ^a^	*P* value
ASC ^b^		0.810	0.064	< 0.01	0.611	0.052	< 0.01
*Attributes*							
1) Patient experiences	*Below average (ref.)*						
	*Average*	0.816	0.087	< 0.01	1.007	0.062	< 0.01
	*Above average*	1.515	0.091	< 0.01	1.217	0.069	< 0.01
2) Clinical outcome indicator							
Breast cancer:	*Tumor-positive resection margin (in %)*	−0.150	0.010	< 0.01			
Cataract:	*Per-operatively performed vitrectomy (in %)*				−2.567	0.130	< 0.01
3) Waiting time	*(In working days)*	−0.058	0.004	< 0.01	− 0.016	0.002	< 0.01
4) Travel distance	*3 km (ref.)*						
	*8 km*	− 0.351	0.066	< 0.01	− 0.181	0.057	< 0.01
	*15 km*	−0.422	0.064	< 0.01	− 0.674	0.072	< 0.01
5) Recommendation	*Nobody (ref.)*						
	*Friends and Family*	0.659	0.077	< 0.01	−0.029	0.053	0.58
	*GP*	1.259	0.088	< 0.01	0.507	0.062	< 0.01
*SD of random parameters*							
1) Patient experiences	*Average*	0.764	0.120	< 0.01	0.227	0.130	0.08
	*Above average*	−0.855	0.114	< 0.01	− 0.857	0.086	< 0.01
2) Clinical outcome indicator							
Breast cancer:	*Tumor-positive resection margin (in %)*	0.190	0.012	< 0.01			
Cataract:	*Per-operatively performed vitrectomy (in %)*				2.679	0.135	< 0.01
3) Waiting time (in working days)		−0.072	0.004	< 0.01	− 0.033	0.003	< 0.01
4) Travel distance	*8 km*				−0.410	0.142	< 0.01
	*15 km*				−1.143	0.108	< 0.01
5) Recommendation	*Friends and Family*	−0.811	0.117	< 0.01	0.280	0.151	0.06
	*GP*	0.530	0.147	< 0.01	−0.783	0.095	< 0.01
Number of individuals		631			1109		
Number of observations		6304			10,980		
Model fit	*Log-likelihood*	− 2920.42			− 4915.45		
	*BIC*	5972.07			9989.06		

All random parameters were assumed to be normally distributed and were simulated using 5000 Modified Latin Hypercube Sampling draws

^a^reflects bootstrapped standard errors

^b^coded as 1 for the first (left) alternative and 0 for the second alternative. The coefficient reflects the utility derived from any given hospital presented on left hand side of the choice set and thus accounted for any left-to-right bias

*ASC* Alternative Specific Constant, *BIC* Bayesian information criterion, *GP* General Practitioner, *KM* Kilometer, *NA* Not Available, *Ref*. Reference, *SD* Standard deviation, *SE* Standard error

Regarding the relative importance of attributes (Table 4), clinical outcome indicator was the most important attribute for both cataract and breast cancer as it yielded the largest MRS. For example, cataract patients were, on average, willing to wait an additional 210.5 working days (95% CI: 140.8–280.2) to be able to select the hospital that had scored 0.4% (i.e., favorable score) on the per-operatively performed vitrectomy’ indicator over a hospital with a 1.7% score (i.e., unfavorable score). For both patient groups, the second most important attribute was patient experiences. The importance of both quality-of-care attributes implied that patients were willing to wait longer for better quality of care. In addition, the least important attribute was travel distance (breast cancer) and recommendation (cataract). The latter was in line with the lower self-reported importance of the advice of GPs among cataract patients relative to breast cancer patients as described above and shown in Table 2. Moreover, the observed MRS values for cataract were larger and less concentrated in terms of range (range: 32.2 to 210.5) relative to those for breast cancer (range: 3.0 to 51.4). This implied that, on average and ceteris paribus, cataract patients were more responsive to a change in level at a given attribute compared to breast cancer patients.

**Table 4:** Marginal rate of substitution (main analyses)

	Breast cancer		Cataract		***Interpretation***
	Marginal willingness to wait (in working days)	95%CI (lower bound; upper bound) ^a^	Marginal willingness to wait (in working days)	95%CI (lower bound; upper bound) ^a^	To select a hospital that …
*Attributes*					
1) Patient experiences	18.6	(14.3–22.9)	77.3	(52.8–101.8)	… scored above average on patient experiences instead of a hospital that scored below average on patient experiences.
2) Clinical outcome indicator	38.6	(32.9–44.2)			… reported a 5% score on the ‘tumor-positive resection margin’ indicator instead of a hospital with a 20% score.
			210.5	(140.8–280.2)	… reported a 0.4% score on the ‘per-operatively performed vitrectomy’ indicator instead of a hospital with a 1.7% score.
4) Travel distance	3.0	(1.1–4.9)	40.6	(25.9–55.3)	… was located 3 km away from their home instead of a hospital located 15 km away.
5) Recommendation	14.6	(10.5–18.7)	32.2	(22.7–41.7)	… was recommended by their GP instead of a hospital that was not recommended by anyone in particular.

^a^computed using robust standard errors

*95%CI* 95% Confidence Intervals, *GP* General Practitioner, *KM* Kilometer, *MRS* Marginal Rate of Substitution

### Predicted and observed distribution of patients

Supplementary Material 2 provides a detailed description of assigned levels for the four general hospitals in real-life settings. In short, for breast cancer and given their assigned levels, hospital 2 and 4 were labelled as “nearby located” hospitals and hospital 3 as the “overall best quality of care” hospital. For cataract, hospital 4 was labelled as a “nearby located” hospital, while hospital 2 was labelled both as a “nearby located” hospital and as the “overall best quality of care” hospital.

**Fig. 1:**
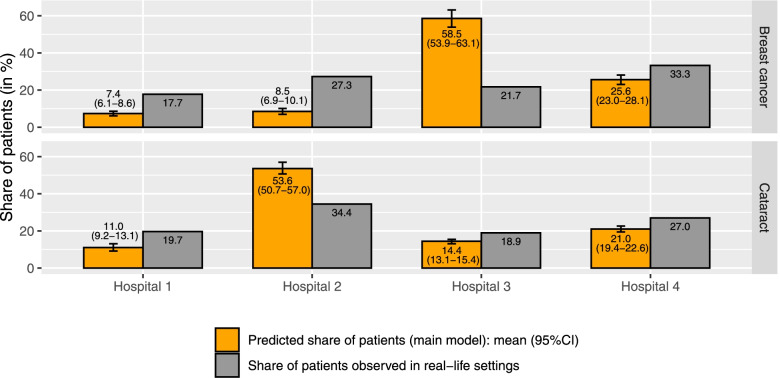
The predicted distribution of patients across the four general hospitals per condition (based on the main analyses) and the observed distribution in real-life settings

As shown in Fig. 1, the predicted distribution of patients across the four hospitals differed from that observed in real-life settings in terms of absolute value and relative order, and these differences were more apparent for breast cancer than for cataract. While most breast cancer patients (average (95%CI)): 58.5% (53.9–63.1)) were expected to select the “best quality of care” hospital (i.e., hospital 3), only 21.7% of the group did so in real-life settings. Instead, most patients selected the hospitals labelled as a “nearby located” hospital (i.e., hospital 2 (27.3%) and hospital 4 (33.3%)). Hence, the relative order of all four hospitals differed between the predicted and observed situation. For cataract, most patients (average (95%CI): 53.6% (50.7–57.0)) were predicted to select hospital 2 (i.e., the “overall best quality of care” hospital). Similar to the predicted situation, the largest share of patients was observed for hospital 2 (34.4%) followed by hospital 4 (27.0%), although proportionally fewer patients selected hospital 2 than expected. Unlike for breast cancer, the relative order of the four hospitals only differed for the two hospitals with smallest share of cataract patients.

### Additional analyses

**Fig. 2:**
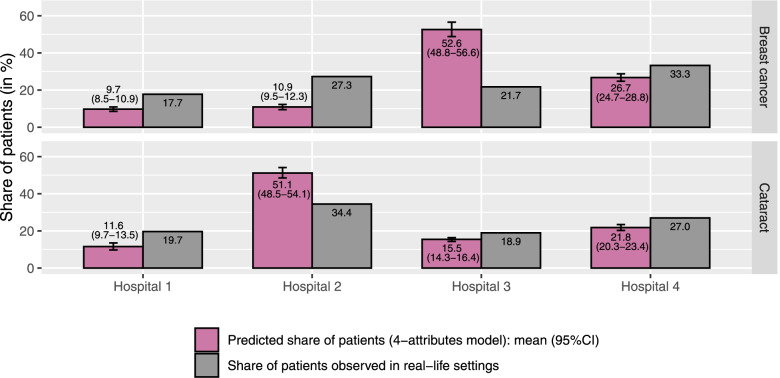
The predicted distribution of patients across the four general hospitals per condition (based on the 4-attributes analyses) and the observed distribution in real-life settings

**Fig. 3:**
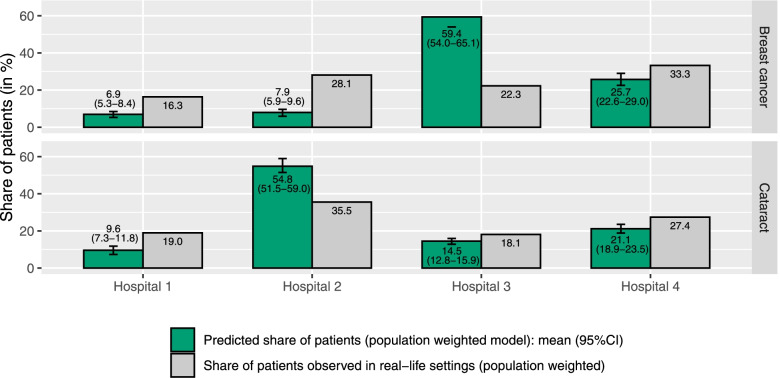
The predicted distribution of patients across the four general hospitals per condition (based on the population weighted analyses) and the population weighted distribution in real-life settings

In the interest of brevity, results of all additional analyses are provided in full detail in Supplementary Material 3. In 4-attributes analyses and in IPW analyses, similar results were observed and did not change the overall conclusion of the main analyses: for both analyses, MRS values resembled those of the main analyses and the observed difference in predicted and observed distribution of patients across the four hospitals persisted (Figs. 2 and 3). Moreover, the assessment of explanatory power revealed that travel distance was the most important attribute in the revealed choices for both conditions: as shown in Table 5, this attribute accounted for 85.5% (breast cancer) and 95.5% (cataract) of the log-likelihood.

**Table 5:** Partial log-likelihood (revealed choices)

Attributes excluded	LL	Difference in LL (relative to full model)	% of total difference	Cumulative %	Relative order
***Breast cancer***					
None (full model)	− 613.866				
Travel distance	−855.918	− 242.052	85.5	85.5	1
Clinical outcome indicator (tumor-positive resection margin)	− 632.560	−18.694	6.6	92.1	2
Patient experiences	− 625.541	−11.675	4.1	96.2	3
Waiting time	− 624.841	−10.975	3.8	100.0	4
***Cataract***					
None (full model)	− 1145.788				
Travel distance	− 1501.405	− 355.617	98.5	98.5	1
Patient experiences	− 1149.633	−3.846	1.1	99.6	2
Waiting time	− 1146.522	−0.735	0.2	99.8	3
Clinical outcome indicator (per-operatively performed vitrectomy)	−1146.474	−0.687	0.2	100.0	4

As suggested by Lancsar et al. [29], we used weights to ensure the weighted sample size was equal across all blocks of the experimental design

*LL* Log-Likelihood

## Discussion

### Principal findings

We set out to assess to what extent a patient’s choice of hospital in real-life settings corresponds with their preferred choice. Our results indicated that (1) while both breast cancer and cataract patients valued quality of care in their stated hospital choice, hospital quality was of lesser importance in their revealed choice. (2) In contrast, a patient’s travel distance was the most important attribute in the choice of hospital in real-life settings for both patient groups. (3) The predicted distribution of patients across the four general hospitals differed from that observed in real-life settings in terms of absolute value and, for breast cancer also in relative order. (4) For both conditions and relative to the main analyses, similar results were observed in both the 4-attributes analyses and in  the population weighted analyses.

### Possible explanations and comparison with literature

The discrepancies between the relative importance of attributes in stated and revealed hospital choices, and between the predicted and revealed distribution of patients are consistent with the literature: patients state certain characteristics as important in hypothetical settings, but act upon others in real-life settings [6]. As described in more detail below, this may be explained by the fact that most patients rely on their GP’s advice in patient choice [5, 6, 31], and that the preferences of patients and the physicians who represent them may often not align [32].

With respect to the DCE, we observed the largest MRS values for both conditions for clinical outcome indicators followed by patient experiences. For instance, breast cancer patients were, on average, willing to wait an additional 38.6 working days (95% CI: 32.9–44.2) to select the hospital with a favorable score of 5% on the tumor-positive resection margin’ indicator over a hospital with a score of 20%. However, while highly valued in the DCE, quality information only played a minor role in the revealed hospital choices; a finding that supports previous research. Faber et al. have concluded that, although valued as important by patients, quality information rarely affects decisions in real-life settings [7].

With respect to the revealed hospital choices, the most important attribute was travel distance: additional analyses showed that this attribute contributed for 85.5% (breast cancer) and 95.5% (cataract) of the model’s log-likelihood. The apparent difference in the relative importance of attributes between stated and revealed hospital choices may explain why the predicted distribution of patients differed from that observed in real-life settings. For breast cancer, while most patients were expected to select the hospital labelled as “best quality of care“, they selected the hospitals labelled as “nearby located”. For cataract, a similar shift was observed, although it did not affect the relative order of hospitals in terms of shares of patients. The smaller shift may be explained by the fact that the hospital labelled as the one with the “best overall quality of care" “was also labelled as a “nearby located” hospital. Our finding that patients generally go to the closest hospital, is also in line with literature: research has shown that (1) patients generally go to the nearest provider, (2) that they prefer the status-quo option (i.e., prefer to be treated in the hospital where they already have been treated), and (3) that they are only more likely to switch hospitals if they have had a bad experience with their current provider or are faced with long waiting lists [5, 6].

While consistent with previous studies, the limited importance of quality information in practice is somewhat surprising in the light of our study protocol [10]. Some of the GPs involved in our study were also involved in the development of the patient report cards [10], and were likely to be motivated to stimulate patient choice while using these cards. Moreover, as part of our study protocol, all GPs were instructed to present patients with these report cards to stimulate patient choice [10]. Hence, patient experiences and clinical outcome indicator were expected to be of greater importance in the revealed hospital choices. The literature provides a possible explanation. Most patients rely on the advice of their choice of hospital [5, 6, 31]. However, research has shown that preferences of patients and those who act on their behalf (e.g., physicians) may often not align [32]. For instance, in a study conducted by Empel et al. physicians have underestimated the importance of patient experiences to patients regarding fertility care [33]. Moreover, studies have shown that GPs generally do not take quality information into account in their referrals, but rather rely on other factors such as their own preferences, close connections and good previous experiences with the specialists working at a given hospital [9–11]. On the one hand and given the shown importance of the GP’s advice, these factors may, ceteris paribus, explain the lower relative impact of quality information observed in our study. On the other hand, it is reasonable to assume that GPs have close connections with the specialists working at nearby hospitals. As shown by the large explanatory power in our study, travel distance may capture the close relationship between the GP and the hospital departments and act as a proxy thereof in practice. This implies that patients are more willing to travel beyond their nearest hospital for better hospital quality than research suggests [5, 6].

### Implications for research and practice

Our study shows that the assumption of many current policy reforms - patients and referring physicians who choose on their behalf, choose hospitals in accordance to the preferences of patients – is unlikely to hold in practice. At the point of referral, other factors (e.g., time constraints) come into play that prevent the patient’s stated preferences to prevail.

For patients to adopt the active role which is often assumed in health policy, additional efforts are required. If patients are in fact willing to take charge in the decision-making process for a hospital, GPs need to incorporate time during their consultations to discuss possible options, ideally, while using decision support tools. This approach would tackle the two main reasons why most patients do not actively choose their providers: (1) the perceived limited degree of choice (e.g. due to the health insurer’s constraints) and (2) the lack of adequate and suitable information to support their choice (e.g. patients are often overwhelmed by the large amount of publicly available quality information [6]). A tailored decision support tool allows the GP to present all possible options given the patient’s health insurance coverage. Similarly, these tools may tailor comparative information to the needs of patients by presenting only a subset of the available quality indicators (i.e., the most important ones) [34]. However, some patients are not willing or are unable to become actively involved in patient choice and simply prefer their GP to decide on their behalf. Therefore, knowledge of the preferences of the given patient group are needed. This calls for patient-group specific preferences studies to gain insights into what subgroups patients prefer, followed by the development of decision support tools that present GPs with patient-group specific information.

Furthermore, the discrepancy between the predicted and observed distribution of patients should not be seen as potential evidence of the (non)-predictive value of DCEs. For such evidence, the context of the given decision requires the decision-maker to have freedom of choice and to be willing to exercise their choice in accordance to their preferences (see, for example, in health care [35]). Our study has investigated whether patients do in fact have the ability to choose their hospitals in accordance to their preferences and unconstrained by external parties.

### Strengths and limitations

As research comparing the stated and revealed choices of the same sample is scarce [6], our study adds new and unique evidence to the literature. From a methodological perspective, the use of the same sample has allowed us to rule out potential differences in preferences between the stated and revealed choices that may result from sample variability. In addition, we have used DCEs to quantify preferences: a meta-analysis concludes that DCEs are able to predict health-related decisions with a moderate accuracy [36]. Furthermore, we have performed IPW analyses to improve the representativeness of our sample; these analyses have not affected our general conclusion, and thus indicate that our findings are representative for the total patient population in terms of gender, age and educational level.

Our main limitation is that we have not been able to model the attribute recommendation in the revealed choices as this information has not been recorded in claims data. Although we have asked respondents in the questionnaire who had recommended the hospital in which they have been treated, we believe that this information cannot be validly collected for the non-chosen hospitals due to, for example, recall bias. As we have relied on the Random Utility Maximization framework in our models, we expect that an omitted attribute may lead to different marginal utility coefficients and estimated MRS of the included attributes in absolute terms, but it should not impact the relative order of attributes. Findings of our 4-attributes analyses support this hypothesis.

Our second limitation relates to the timing of our study. Regarding the data, we have used data that originates from 2010, 4 years after the 2006’s reform that has introduced provider competition in the Dutch health care system. Patients and GPs may have required more time to fully adapt to their new role with regard to patient choice. However, as our findings are consistent with previous studies that have been conducted across various health systems and time periods [5, 6, 31], we expect that our conclusions still hold in current daily practice. Regarding the experimental design, our design (i.e., a fold-over of an orthogonal main effects plan, forced choice sets) has been constructed in accordance with the common practice of 2010 [37]. Given that both MXL models (breast cancer and cataract) have demonstrated theoretical validity and in accordance with Rose and Bliemer who have stated that “given large enough samples, the underlying experimental design should not matter in terms of statistical power” (p612) [38], we believe that the use of a fold-over design does not affect our findings.

Furthermore, although we have considered as many patient characteristics as possible in our IPW analyses, we do not have data on health status. We expect that patients in poor health may be less likely to participate and thus may be underrepresented in our samples. Similarly, we lack any information on variables such as patient activation [39]. As patients with higher activation levels are more likely to actively choose providers than those with lower levels [6], we hypothesize that, for example, recommendation may play a smaller role in patient choice among the former relative to the latter.

## Conclusion

Our findings show that there is a consistent discrepancy between what patients valued the most in stated hospital choices and in revealed hospital choices. Quality information were valued as important in the DCE, but only played a small role in the revealed hospital choice. We interpret this finding as the result of the patients’ strong dependency on the advice of their GPs who may prefer to refer their patients to hospitals with whom they have close connections and good previous experiences. In practice, this may most likely be the closest hospital. We therefore conclude that patients are more willing to travel further than the nearest hospital for better hospital quality than is often assumed in the literature.

## Supplementary Information

**Additional file 1: Supplementary Material 1.** Example choice set, DCE breast cancer.

**Additional file 2: Supplementary Material 2.** Hospital characteristics (attributes) and levels, the four general hospitals.

**Additional file 3: Supplementary Material 3.** Results of additional analyses.

## Data Availability

The datasets used and/or analyzed during this study are available from the corresponding author on reasonable request.

## Abbreviations

ASC: Alternative Specific Constant
BIC: Bayesian Information Criterion
CQI: Consumer Quality Index
DCE: Discrete choice experiment
GP: General Practitioner
IPW: Inverse probability weighted
KM: Kilometer
LL: Log-Likelihood
MNL: Multinomial logit
MRS: Marginal Rate of Substitution
MXL: Mixed logit
NA: Not Available
R2: R-squares
SD: Standard deviation
SE: Standard error
US: United States
95%CI: 95% confidence intervals
