# Supplementary material for: Do patients’ preferences prevail in hospital selection?: a comparison between discrete choice experiments and revealed hospital choice
Source: BMC Health Serv Res. 2022 Sep 8;22:1136. doi: 10.1186/s12913-022-08403-6 (PMC9461248; doi:10.1186/s12913-022-08403-6)
Supplement: Supplementary file 1 — Additional file 1: Supplementary Material 1. Example choice set, DCE breast cancer. [file 12913_2022_8403_MOESM1_ESM.pdf]

SUPPLEMENTARY MATERIAL 1.

Example choice set, DCE breast cancer

Imagine the following situation:

- You have been told by your GP you need hospital care to treat breast cancer.
- You may only choose between hospital A or hospital B.
- Both hospitals are practically identical (e.g., travelling facilities options such as a bus stop located nearby, and the number of parking places are the same for both hospitals), and thus differ only in characteristic presented.

For each situation:

1) Compare both hospitals according to each attribute presented.
2) Given the information presented, in which hospital would you like to be treated?
3) Please indicate at the bottom in which hospital would you prefer to be treated.

It is important to note that your decision is never considered right or wrong.

Hospital:	**Hospital A**	**Hospital B**
**1. Patient experiences**<br>The level that other patients are satisfied with the attention, explanation and time received from the hospital staff.<br>Higher values reflect better scores.	Below average	Average
**2. Clinical outcome indicator**<br>An indicator of hospital quality that reflects the share of resections for which the tumor resection margin was shown to be tumor-positive in the first surgery in breast saving therapy.<br>Lower values reflect better scores.	10%	5%
**3. Waiting time**<br>The number of working days you have to wait until the first appointment.	5 working days	15 working days
**4. Travel distance**<br>The number of kilometers from your home to the hospital.	15 kilometers	8 kilometers
**5. Recommendation**<br>The person or persons who recommends you the given hospital.	Nobody in particular	Your General Practitioner
In which hospital would you prefer to be treated?		
