# Supplementary material for: Do patients’ preferences prevail in hospital selection?: a comparison between discrete choice experiments and revealed hospital choice
Source: BMC Health Serv Res. 2022 Sep 8;22:1136. doi: 10.1186/s12913-022-08403-6 (PMC9461248; doi:10.1186/s12913-022-08403-6)
Supplement: Supplementary file 2 — Additional file 2: Supplementary Material 2. Hospital characteristics (attributes) and levels, the four general hospitals. [file 12913_2022_8403_MOESM2_ESM.pdf]

SUPPLEMENTARY MATERIAL 2.

Hospital characteristics (attributes) and levels, the four general hospitals

Table 6 shows the combination of assigned levels based on the hospital's scores on corresponding indicators and the patient-hospital distance in real-life settings. Relative to the other hospitals, hospital 2 had the second smallest waiting time and scored, together with hospital 4, the best in terms of travel distance: for both hospitals, 54.4% of the patients had been assigned with the furthest travel-distance level (i.e., "15 kilometers" level) which indicated that fewer patients had to travel this distance to reach these hospitals compared to hospital 1 (75.1%) and hospital 3 (66.5%). Hospital 3 reported the most favorable scores on two attributes (patient experiences and waiting time), while its score on clinical outcome indicator came close to the best score achieved by hospital 4. Given their assigned levels, hospital 2 and 4 were labelled as "nearby located" hospitals and hospital 3 as the "best quality of care" hospital.

With respect to cataract, hospital 1 reported the best score on patient experiences relative to the other hospitals, but scored poorly on the other attributes. Hospital 2, 3 and 4 had similar scores on clinical outcome indicator, although hospital 4's score was slightly better. Hospital 2 and 4 had favorable scores for both waiting time and travel distance. Hospital 3 and 4 scored below average on patient experiences. Considering their assigned levels, hospital 4 was labelled as a "nearby located" hospital, while hospital 2 was labelled both as a "nearby located" and as the "overall best quality of care" hospital.

Table 6. Hospital characteristics (attributes) and levels, the four general hospitals in real-life settings

Hospital		1	2	3	4
**Breast cancer**					
Assigned levels on each attribute					
1. Patient experiences [a]		Average	Average	Above average	Average
2. Clinical outcome indicator	Tumor-positive resection margin	12.8%	13.7%	5.6%	4.8%
3. Waiting time		7 working days	3 working days	2 working days	7 working days
4. Travel distance [b]	Share of patients: 3 kilometers	8.9%	19.3%	10.0%	17.3%
	8 kilometers	16.0%	26.3%	23.5%	28.4%
	15 kilometers	75.1%	54.4%	66.5%	54.4%
5. Recommendation		NA	NA	NA	NA
Assigned hospital label					
Label		(none)	Nearby located	Best quality of care	Nearby located

Table 6. Hospital characteristics (attributes) and levels, the four general hospitals in real-life settings *(continued)*

Hospital		1	2	3	4
**Cataract**					
Assigned levels on each attribute					
1. Patient experiences [a]		Above average	Average	Below average	Below average
2. Clinical outcome indicator	Per-operatively performed vitrectomy	0.79%	0.44%	0.44%	0.41%
3. Waiting time		66 working days	14 working days	28 working days	14 working days
4. Travel distance [b]	Share of patients:<br>3 kilometers	9.5%	18.8%	5.9%	16.6%
	8 kilometers	12.0%	22.2%	20.3%	24.3%
	15 kilometers	78.5%	59.0%	73.9%	59.1%
5. Recommendation		NA	NA	NA	NA
Assigned hospital label					
Label		(none)	Nearby located, and overall best quality of care	(none)	Nearby located

[a] = based on the Dutch CQI questionnaire (a translated and validated version of the Consumer Assessment of Healthcare Providers and Systems implemented and used in the US [22]). b = the assigned level for a given hospital differed across patients. For example, the travel distance from the patient's home to hospital 1 was equal to 3 kilometers or less for 56 breast cancer patients (8.9%), between 3 and 8 kilometers for 101 patients (16.0%), and more than 8 kilometers (i.e., "15 kilometer" level) most patients (n=474, 75.1%).

NA = Data was not available in claims data.
