# Supplementary material for: Do patients’ preferences prevail in hospital selection?: a comparison between discrete choice experiments and revealed hospital choice
Source: BMC Health Serv Res. 2022 Sep 8;22:1136. doi: 10.1186/s12913-022-08403-6 (PMC9461248; doi:10.1186/s12913-022-08403-6)
Supplement: Supplementary file 3 — Additional file 3: Supplementary Material 3. Results of additional analyses. [file 12913_2022_8403_MOESM3_ESM.pdf]

SUPPLEMENTARY MATERIAL 3

Results of additional analyses

Table 7. Results of mixed logit models (4-attributes analyses)

		Breast cancer			Cataract		
		Beta	SE [a]	P value	beta	SE [a]	P value
ASC [b]		0.607	0.050	<0.01	0.553	0.046	<0.01
Attributes							
1) Patient experiences	Below average (ref.)						
	Average	0.610	0.066	<0.01	0.912	0.054	<0.01
	Above average	1.172	0.062	<0.01	1.058	0.059	<0.01
2) Clinical outcome indicator							
Breast cancer:	Tumor-positive resection margin (in %)	-0.123	0.007	<0.01			
Cataract:	Per-operatively performed vitrectomy (in %)				-2.305	0.104	<0.01
3) Waiting time	(In working days)	-0.047	0.003	<0.01	-0.015	0.002	<0.01
4) Travel distance	3 km (ref.)						
	8 km	-0.231	0.054	<0.01	-0.138	0.051	<0.01
	15 km	-0.304	0.053	<0.01	-0.606	0.065	<0.01
5) Recommendation	Nobody (ref.)						
	Friends and Family						
	GP						

SD of random parameters							
1) Patient experiences	Average	-0.538	0.104	<0.01	0.068	0.075	0.36
	Above average	-0.492	0.127	<0.01	-0.763	0.077	<0.01
2) Clinical outcome indicator							
Breast cancer:	Tumor-positive resection margin (in %)	0.167	0.009	<0.01			
Cataract:	Per-operatively performed vitrectomy (in %)				2.367	0.112	<0.01
3) Waiting time (in working days)		-0.061	0.003	<0.01	-0.028	0.003	<0.01
4) Travel distance	8 km				-0.351	0.139	0.01
	15 km				-1.037	0.095	<0.01
5) Recommendation	Friends and Family						
	GP						
Number of individuals		631			1109		
Number of observations		6304			10980		
Model fit	Log-likelihood	-3084.83			-5002.35		
	BIC	6265.90			10125.65		

All random parameters were assumed to be normally distributed and were simulated using on 5,000 Modified Latin Hypercube Sampling draws.
[a] = reflects bootstrapped standard errors. [b] = coded as 1 for the first (left) alternative and 0 for the second alternative. The coefficient reflects the utility derived from any given hospital presented on left hand side of the choice set and thus accounted for any left-to-right bias.
ASC = alternative specific constant. BIC = Bayesian information criterion. GP = General Practitioner. KM = kilometer. NA = not available. Ref. = Reference. SD = Standard deviation. SE = Standard error.

Table 8. Marginal rate of substitution (4-attributes analyses)

	Breast cancer		Cataract		Interpretation
	Marginal willingness to wait (in working days)	95%CI (lower bound; upper bound) [a]	Marginal willingness to wait (in working days)	95%CI (lower bound; upper bound) [a]	To select a hospital that…
Attributes					
1) Patient experiences	15.8	(12.8-18.9)	73.0	(55.0-91.0)	… scored above average on patient experiences instead of a hospital that scored below average on patient experiences.
2) Clinical outcome indicator	38.8	(33.3-44.2)			… reported a 5% score on the 'tumor-positive resection margin' indicator instead of a hospital with a 20% score.
			204.5	(155.6-253.3)	… reported a 0.4% score on the 'per-operatively performed vitrectomy' indicator instead of a hospital with a 1.7% score.
4) Travel distance	3.5	(1.2-5.8)	39.1	(27.3-50.9)	… was located 3 km away from their home instead of a hospital located 15 km away.
5) Recommendation					… was recommended by their GP instead of a hospital that was not recommended by anyone in particular.

[a] = computed using robust standard errors.
95%CI = 95% Confidence Intervals. GP = General Practitioner. KM = kilometer. MRS = Marginal Rate of Substitution.

Table 9. Results of mixed logit models (IPW analyses)

		Breast cancer			Cataract		
		beta	SE [a]	P value	beta	SE [a]	P value
ASC [b]		0.826	0.067	<0.01	0.580	0.067	<0.01
Attributes							
1) Patient experiences	Below average (ref.)						
	Average	0.826	0.100	<0.01	1.045	0.087	<0.01
	Above average	1.529	0.106	<0.01	1.308	0.096	<0.01
2) Clinical outcome indicator							
Breast cancer:	Tumor-positive resection margin (in %)	-0.160	0.013	<0.01			
Cataract:	Per-operatively performed vitrectomy (in %)				-3.222	0.228	<0.01
3) Waiting time	(In working days)	-0.058	0.004	<0.01	-0.016	0.002	<0.01
4) Travel distance	3 km (ref.)						
	8 km	-0.357	0.071	<0.01	-0.170	0.071	0.02
	15 km	-0.381	0.072	<0.01	-0.671	0.096	<0.01
5) Recommendation	Nobody (ref.)						
	Friends and Family	0.669	0.091	<0.01	-0.068	0.070	0.33
	GP	1.247	0.102	<0.01	0.575	0.089	<0.01

SD of random parameters							
1) Patient experiences	Average	-0.800	0.131	<0.01	-0.327	0.170	0.05
	Above average	0.784	0.136	<0.01	-0.854	0.119	<0.01
2) Clinical outcome indicator							
Breast cancer:	Tumor-positive resection margin (in %)	0.201	0.014	<0.01			
Cataract:	Per-operatively performed vitrectomy (in %)				3.071	0.207	<0.01
3) Waiting time (in working days)		-0.071	0.005	<0.01	-0.026	0.003	<0.01
4) Travel distance	8 km				0.042	0.114	0.71
	15 km				1.134	0.135	<0.01
5) Recommendation	Friends and Family	0.875	0.137	<0.01	0.061	0.138	0.66
	GP	-0.519	0.159	<0.01	-0.907	0.113	<0.01
Number of individuals		631			1109		
Number of observations		6304			10980		
Model fit	Log-likelihood	-2889.21			-4514.39		
	BIC	5909.65			9186.95		

All random parameters were assumed to be normally distributed and were simulated using on 5,000 Modified Latin Hypercube Sampling draws. [a] = reflects bootstrapped standard errors. [b] = coded as 1 for the first (left) alternative and 0 for the second alternative. The coefficient reflects the utility derived from any given hospital presented on left hand side of the choice set and thus accounted for any left-to-right bias.
ASC = alternative specific constant. BIC = Bayesian information criterion. GP = General Practitioner. KM = kilometer. NA = not available. Ref. = Reference. SD = Standard deviation. SE = Standard error.

Table 10. Marginal rate of substitution (IPW analyses)

	Breast cancer		Cataract		Interpretation
	Marginal willingness to wait (in working days)	95%CI (lower bound; upper bound) [a]	Marginal willingness to wait (in working days)	95%CI (lower bound; upper bound) [a]	To select a hospital that…
Attributes					
1) Patient experiences	18.4	(13.7-23.1)	84.1	(66.0-102.2)	… scored above average on patient experiences instead of a hospital that scored below average on patient experiences.
2) Clinical outcome indicator	41.1	(34.4-47.9)			… reported a 5% score on the 'tumor-positive resection margin' indicator instead of a hospital with a 20% score.
			265.5	(214.6-316.3)	… reported a 0.4% score on the 'per-operatively performed vitrectomy' indicator instead of a hospital with a 1.7% score.
4) Travel distance	2.6	(0.2-5.0)	42.3	(27.7-56.8)	… was located 3 km away from their home instead of a hospital located 15 km away.
5) Recommendation	14.9	(10.1-19.6)	36.6	(23.5-49.6)	… was recommended by their GP instead of a hospital that was not recommended by anyone in particular.

[a] = computed using robust standard errors.
95%CI = 95% Confidence Intervals. GP = General Practitioner. KM = kilometer. MRS = Marginal Rate of Substitution.
